# Unexplained Bilateral Adrenal Hemorrhage and Adrenal Insufficiency Unveiling Burkitt's Lymphoma

**DOI:** 10.1016/j.aed.2025.03.007

**Published:** 2025-04-10

**Authors:** Majd Oweidat, Anas Abu Rumilah, Mohammed Maraqa, Motaz Natsheh

**Affiliations:** 1 College of Medicine, Hebron University, Hebron, West Bank, Palestine; 2 Division of Endocrinology, Department of Internal Medicine, Al-Ahli Hospital, Hebron, West Bank, Palestine; 3 Department of Surgery, Al-Ahli Hospital, Hebron, West Bank, Palestine; 4 Department of Surgery, Bethlehem Arab Society for Rehabilitation (BASR), Bethlehem, West Bank, Palestine; 5 Department of Surgery, Princess Alia Hebron Governmental Hospital, Hebron, West Bank, Palestine; 6 Department of Pathology, Al-Ahli Hospital, Hebron, West Bank, Palestine

**Keywords:** adrenal hemorrhage, adrenal insufficiency, Burkitt lymphoma, malignancy, lymphoma

## Abstract

**Background/Objective:**

Adrenal hemorrhage (AH) and adrenal insufficiency (AI) are rare, life-threatening conditions, typically linked to trauma, sepsis, or anticoagulation. Burkitt's lymphoma (BL), an aggressive B-cell non-Hodgkin lymphoma, rarely involves the adrenal glands and has not been reported to present with AH and AI. This report describes a unique case of AH and AI presenting as manifestations of BL, highlighting the importance of considering malignancy in unexplained AH and AI.

**Case Report:**

A 16-year-old boy presented with acute right flank pain and muscle spasms. Imaging revealed a right suprarenal hematoma with active contrast extravasation, managed with adrenal artery embolization. Two weeks later, he developed recurrent abdominal pain, vomiting, and distention. Computed tomography showed bilateral adrenal enlargement with necrosis and hemorrhage. Laboratory test results revealed normocytic anemia, increased lactate dehydrogenase level, and hypoglycemia. AI was confirmed (serum cortisol level, 1.5 μg/dL; adrenocorticotropic hormone level, 82 pg/mL), prompting hydrocortisone replacement. Positron emission tomography/computed tomography revealed hypermetabolic lesions in bilateral adrenal glands, lymph nodes, pleura, peritoneum, and bone marrow. Neck lymph node biopsy confirmed BL, showing a “starry sky” pattern, CD20 positivity, and Ki-67 nearing 100%. Intensive chemotherapy led to significant clinical improvement.

**Discussion:**

This case highlights the diagnostic challenge of AH and AI without typical risk factors. BL, although rarely involving the adrenal glands, should be considered in unexplained AH.

**Conclusion:**

This report highlights the importance of considering malignancy in unexplained AH and AI, even without conventional risk factors.

**Highlights**

• Burkitt lymphoma (BL) presenting with adrenal hemorrhage (AH) and adrenal insufficiency (AI) is unprecedented
• AH and AI in BL highlight the need to consider malignancy in unexplained adrenal pathology
• BL should be included in the differential diagnosis of AH, even without typical risk factors

**Clinical Relevance**

This case highlights the importance of considering malignancy, such as Burkitt lymphoma, in patients presenting with unexplained adrenal hemorrhage and adrenal insufficiency.

## Introduction

Adrenal hemorrhage (AH) is an uncommon and potentially life-threatening condition that typically presents with nonspecific symptoms. It can lead to adrenal crisis, shock, or even death.^1^ Although most cases of AH result from abdominal trauma and involve a single adrenal gland, bilateral AH (BAH) represents approximately 10% of all cases and is more frequently associated with systemic conditions such as coagulopathies, septicemia, or surgery.^2^ BAH has critical clinical implications because it often causes adrenal insufficiency (AI) when >90% of the adrenal cortex is affected.^3^ Beyond systemic conditions, the underlying causes of BAH vary widely and include trauma, infections, antiphospholipid syndrome (APS), anticoagulant use, and neoplasms.^2^

Neoplastic causes of AH, such as primary adrenal tumors or metastatic disease, are particularly rare; however, their recognition is crucial in patients presenting with adrenal masses, hemorrhage, or signs of AI.^2^ Pheochromocytomas are the most common type of tumors associated with AH.^4^

Burkitt lymphoma (BL) is an aggressive B-cell non-Hodgkin lymphoma that frequently involves extranodal sites.^5^ It accounts for 1% to 5% of all non-Hodgkin lymphomas and shows a strong male predominance, with a male-to-female ratio of 3 to 4:1.^6^ In pediatric populations, BL is relatively common, with an annual incidence of 3 to 6 cases per 100 000 children. The mean age at diagnosis for pediatric patients typically ranges from 3 to 12 years.^5^

Here, we report a case of unexplained AH and AI, which ultimately led to the diagnosis of BL, suggesting a potential causal relationship between these conditions.

## Case Report

A 16-year-old male patient with no significant medical history presented with acute-onset right flank pain and muscle spasms. The pain was described as intermittent and unresponsive to analgesics. Initial investigations at another facility revealed anemia, and a computed tomography (CT) scan identified a right suprarenal hematoma with active contrast extravasation. The patient subsequently underwent right adrenal artery embolization and was discharged after a 7-day hospitalization in stable condition. Two weeks later, he experienced a recurrence of generalized abdominal pain, more severe on the right side, accompanied by vomiting and abdominal distention. Notably, there was no history of fever, night sweats, weight loss, trauma, or anticoagulant use. He was referred to our surgical intensive care unit for further evaluation and management.

On admission, the patient appeared hemodynamically stable but was tachycardic with a blood pressure of 140/90 mm Hg, a respiratory rate of 19 breaths per minute, and oxygen saturation of 96% on room air. Abdominal examination revealed significant tenderness in the right flank, whereas cardiovascular, respiratory, and neurologic examinations were unremarkable. No palpable lymph nodes or masses were detected in the head or neck regions.

**Fig. 1:**
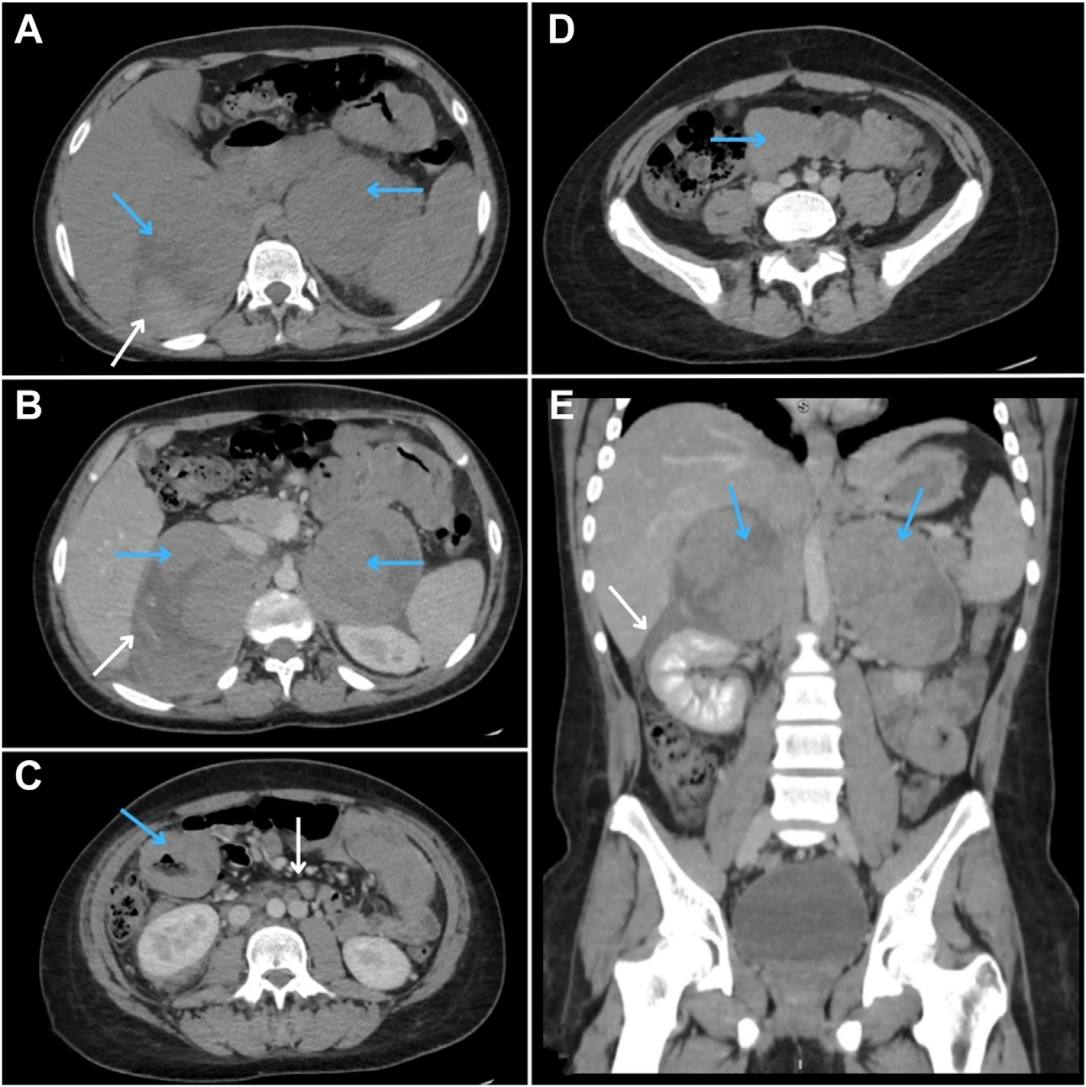
Axial and coronal abdomen computed tomography (CT) scan with and without intravenous contrast. *A*, Axial CT image showed a large, well-defined heterogeneous collections (hematomas) in both adrenal glands (blue arrows) with peripheral dense nodules of soft tissue density (white arrows). *B*, Postcontrast imaging revealed enhancement of the peripheral dense regions of the hematomas (blue arrows) and active contrast extravasation within the right adrenal hematoma (white arrow). *C, D*, Diffuse enhancing wall thickening of small bowel loops was evident (blue arrows), alongside multiple enlarged para-aortic lymph nodes (white arrows). A duplicated inferior vena cava was observed as an incidental finding. *E*, Coronal CT images displayed bilateral adrenal hematomas (blue arrows) with peripheral enhancing nodules and minor extension into the perinephric space on the right side (white arrow).

Initial abdominal contrast-enhanced CT imaging shown in Figure 1 showed markedly enlarged, heterogeneous bilateral adrenal glands with mild enhancement, areas of necrosis, and active contrast extravasation (right adrenal gland, 12 × 8 × 10 cm; left adrenal gland, 8.5 × 8.3 × 10 cm), suggesting a BAH.

Laboratory evaluations shown in the Table revealed normocytic anemia, thrombocytosis, and mild leukocytosis. Additionally, the levels of inflammatory markers increased. To investigate potential underlying causes of AH, a comprehensive diagnostic workup was performed. Rheumatologic assessment ruled out APS as a common cause, whereas infectious etiologies, including disseminated infections, meningococcemia, and sepsis, were systematically excluded. A hemolysis profile showed an increased lactate dehydrogenase level, a low haptoglobin level, and an increased reticulocyte count. The Coombs test results were negative. Coagulation studies showed unremarkable findings. Additionally, biochemical evaluations revealed normal renal and liver function, along with normal electrolytes, whereas urinalysis showed trace protein and ketones.

**Table:** Laboratory Findings in a Patient With Adrenal Hemorrhage and Adrenal Insufficiency

Parameter	Value	Normal Range
Hemoglobin	9.3 g/dL	13.0-16.0 g/dL
Platelet count	387 × 10^9^/L	150-450 × 10^9^/L
White blood cell	9.4 × 10^9^/L	4.5-13.5 × 10^9^/L
Neutrophils	68%	35%-65%
Lymphocytes	20%	25%-45%
Monocytes	9.6%	2%-10%
Erythrocyte sedimentation rate	45 mm/h	<15 mm/h
C-reactive protein	31 mg/L	<5 mg/L
Lactate dehydrogenase	1,524 U/L	120-300 U/L
Haptoglobin	<8 mg/dL	30-200 mg/dL
Reticulocyte count	0.0406	0.5%-1.5%
Coombs tests	Negative	Negative
Fibrinogen	420 mg/dL	200-400 mg/dL
D-Dimer	3523 ng/mL	<500 ng/mL
Creatinine	0.5 mg/dL	0.4-1 mg/dL
Sodium	141 mmol/L	135-145 mmol/L
Potassium	3.5 mmol/L	3.5-5.5 mmol/L
Calcium	8.4 mg/dL	8.8-10.8 mg/dL
Serum glucose	107 mg/dL	70-140 mg/dL (random)
Albumin	3.5 g/dL	3.5-5.5 g/dL
Urine protein	+1	Negative
Urine ketones	+1	Negative
Antinuclear antibody	Negative	Negative
Extractable nuclear antigen	Negative	Negative
Beta-2 glycoprotein	Negative	Negative
Anticardiolipin antibodies	Negative	Negative
Anti–citrullinated protein antibody	Negative	Negative
Antiphospholipid antibodies	Negative	Negative
Lupus anticoagulant	Negative	Negative
Human immunodeficiency virus	Negative	Negative
Cytomegalovirus immunoglobulin G	Positive	Positive
Epstein-Barr virus immunoglobulin G	Positive	Positive
Procalcitonin	Negative	<0.05 ng/mL
Hepatitis B and C	Negative	Negative
International normalized ratio	1.1	0.8-1.2
Activated partial thromboplastin time	20 s	25-35 s
Prothrombin time	13.6 s	11-15 s
Bleeding time	4 min	2-7 min
Serum cortisol	2 μg/dL	5-23 μg/dL
Fasting cortisol	1.5 μg/dL	5-23 μg/dL
Adrenocorticotropic hormone	82 pg/mL	10-60 pg/mL
Creatine phosphokinase	37 U/L	22-200 U/L
Uric acid	9 mg/dL	3.5-7.2 mg/dL

This table summarizes the key laboratory results obtained during the diagnostic evaluation of the patient, which were essential in excluding common causes of adrenal hemorrhage and confirming adrenal insufficiency.

During hospitalization, the patient’s hemoglobin level decreased further from 9.3 to 7.8 g/dL, requiring transfusion of 2 units of packed red blood cells. He developed worsening abdominal distention, increased pain, and intermittent hypotension (blood pressure, 100/50 mm Hg) accompanied by hypoglycemia (blood glucose level, 50 mg/dL). Due to the patient’s worsening clinical condition and unexplained AH, further evaluation was initiated. Endocrine consultation was sought due to suspicion of AI. The low serum cortisol level was 2 μg/dL, the fasting cortisol level was 1.5 μg/dL, and the adrenocorticotropic hormone level was 82 pg/mL, confirming the diagnosis of AI. The patient was started on intravenous *hydrocortisone* (100 mg 3 times daily), leading to stabilization of blood pressure and glucose levels. A surgical consultation deemed bilateral adrenalectomy unnecessary, and a nasogastric tube was inserted for suspected intestinal obstruction.

Follow-up imaging included neck CT that revealed a well-defined mass lesion (1.3 × 1.7 cm) in the right submandibular region, consistent with a calcified lymph node. A chest CT scan showed mild right-sided pleural effusion with collapse consolidation of the surrounding lung but no mediastinal lymphadenopathy or pericardial effusion. An abdominal CT scan revealed mild progression of intra-abdominal free fluid.

**Fig. 2:**
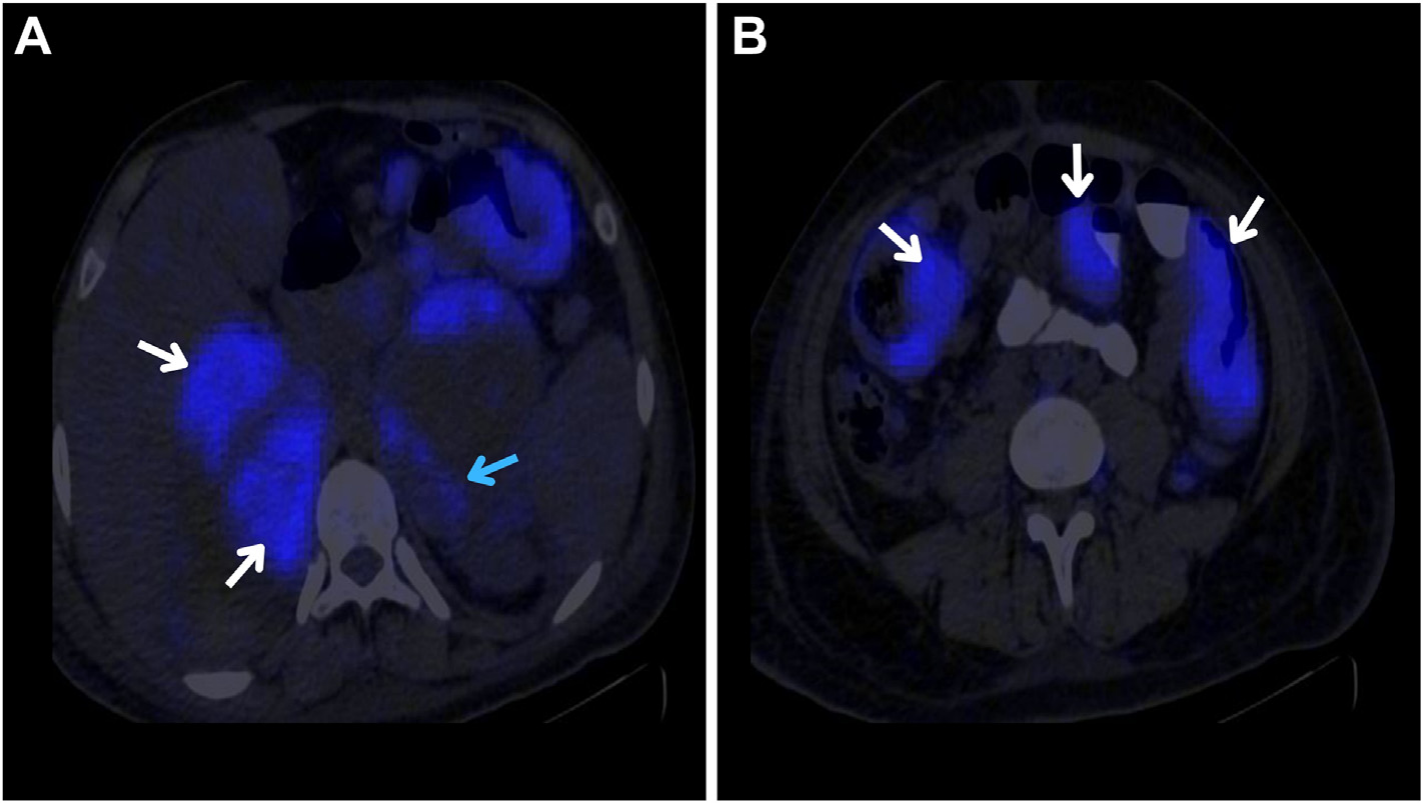
Positron emission tomography–computed tomography imaging of the abdomen in an axial plane. *A*, Fluorodeoxyglucose avid uptake was observed in peripheral dense nodules of adrenal hematomas (white arrow) and in multiple enlarged para-aortic lymph nodes (blue arrow). *B*, Fluorodeoxyglucose avid uptake was noted in diffuse wall thickening involving segments of small bowel loops (white arrow).

On day 4 of admission, the patient developed a bilateral painless submandibular swelling. Examination revealed multiple enlarged lymph nodes in the anterior and posterior triangles of the neck bilaterally. A positron emission tomography/CT scan shown in Figure 2 showed a hypermetabolic, widespread process involving bilateral adrenal glands, lymph nodes above the diaphragm, right pleural deposits, small bowel, peritoneum, and bone marrow, suggesting a systemic malignant process.

**Fig. 3:**
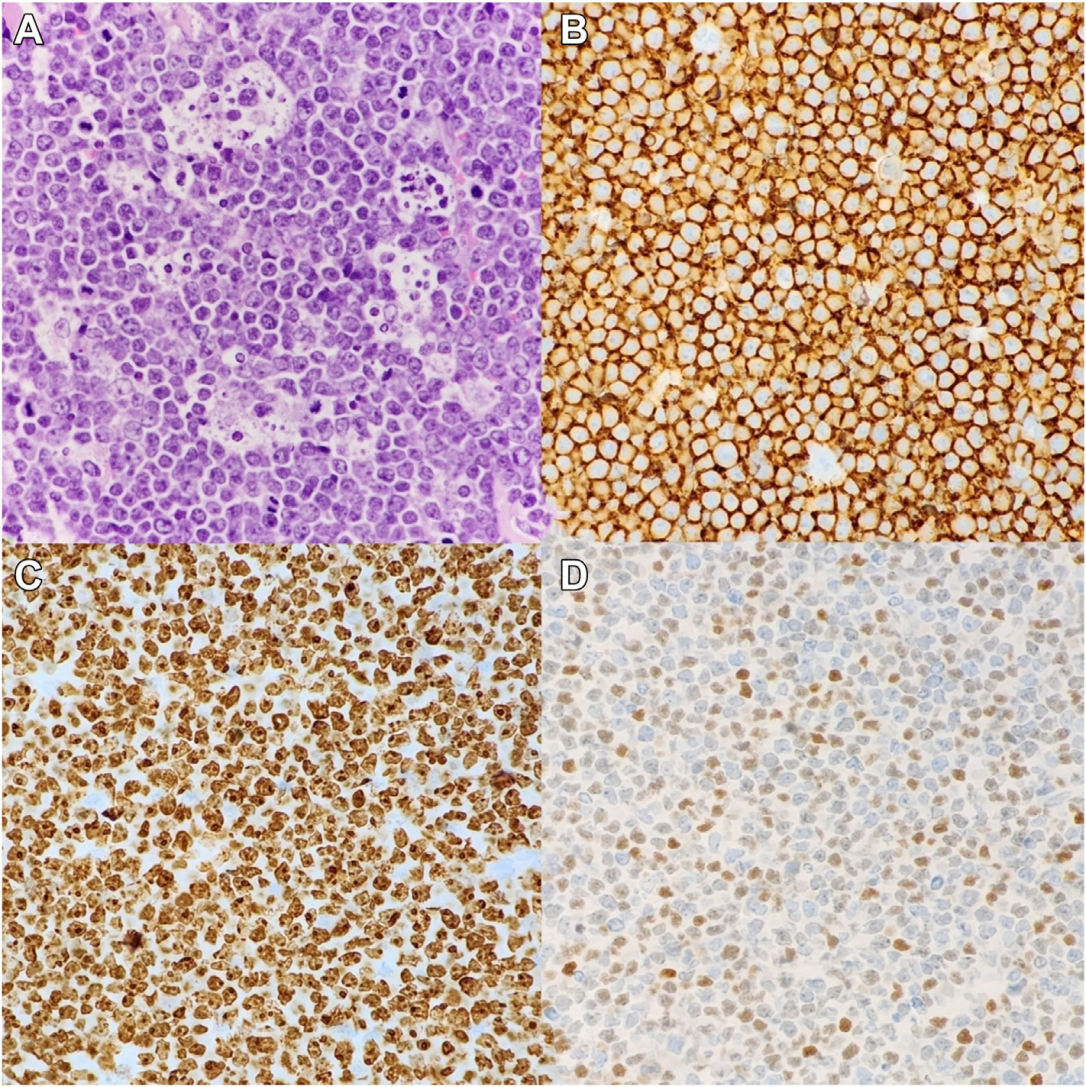
Histopathological and immunohistochemical examination of the submandibular mass biopsy. *A*, Hematoxylin and eosin staining at a magnification of ×40 revealed diffuse proliferation of atypical lymphoid cells with medium-sized, monotonous nuclei, frequent mitotic figures, apoptotic bodies, and scattered tingible body macrophages, displaying a classic “starry sky” appearance. *B*, Immunohistochemical staining showed diffuse positivity for CD20, confirming the B-cell lineage of the neoplastic cells. *C*, Ki-67 immunostaining showed a proliferation index of nearly 100%, indicating the highly proliferative nature of the tumor. *D*, C-MYC immunostaining highlighted positivity in approximately 50% of the tumor cells. Tumor cells were negative for BCL2, TdT, and CD3.

CT-guided excisional biopsy of the largest neck lymph node confirmed the diagnosis of *BL*. The histopathologic examination shown in Figure 3 revealed a classic “starry sky” appearance, characterized by medium-sized monotonous lymphoid cells with high mitotic activity and tingible body macrophages. Immunohistochemical staining confirmed the B-cell lineage with CD20 positivity, a Ki-67 proliferation index nearing 100%, and focal C-MYC positivity. Tumor cells were negative for BCL2, TdT, and CD3. Additionally, a CT-guided biopsy of the right adrenal lesion confirmed the same diagnosis.

The patient was subsequently referred to the hematology-oncology team within <48 hours of diagnosis for treatment and commenced an intensive chemotherapy regimen. The patient showed significant clinical improvement following chemotherapy. Follow-up imaging showed a dramatic reduction in disease burden. AI was managed with ongoing corticosteroid replacement therapy, and the patient achieved clinical stability with supportive care.

## Discussion

This article presents an unusual and diagnostically challenging case of a 16-year-old boy presenting with AH and AI, ultimately attributed to BL, a malignancy not previously presented with AH or AI in the literature.

BL, an aggressive B-cell non-Hodgkin lymphoma, is marked by the t(8;14) translocation, activating the C-MYC oncogene and driving cell proliferation. BL is linked to Epstein-Barr virus, human immunodeficiency virus, and chromosomal translocations.^5^ It has following 3 forms: (1) sporadic (affecting the abdomen/pelvis), (2) endemic (involving facial bones), and (3) immunodeficiency-associated.^5^ BL commonly presents as an abdominal mass, often causing gastrointestinal symptoms such as bowel obstruction. The abdomen is the most frequent site of involvement in sporadic BL, although the head and neck may also be affected. However, our patient initially presented with acute right flank pain, muscle spasms, and unexplained AH, without constitutional symptoms or a detectable abdominal mass. Increased lactate dehydrogenase and uric acid levels are typical due to the rapid tumor growth. Rarely, BL affects other areas such as the mediastinum, central nervous system, or testes, and its presentation in adults often includes constitutional symptoms such as fever and weight loss.^5^ The delayed onset of lymphadenopathy in our patient further deviates from the usual presentation, where generalized lymph node enlargement is often an early finding. Additionally, this case is particularly unusual because AH and AI were a manifestation of BL, an association not previously reported in the literature.

The adrenal glands are particularly susceptible to hemorrhage due to their unique vascular anatomy. The 3 adrenal arteries transition abruptly into capillaries within the zona reticularis, which then converge into a single vein. This structure makes the glands vulnerable to increased capillary pressure if vasoconstriction or thrombotic occlusion of the adrenal vein occurs, often leading to hemorrhage.^2^ AH can lead to AI, especially in patients who are critically ill, with sepsis being a rare but recognized cause.^7^ A study shows that AH occurs in approximately 1.1% of patients with sepsis, resulting in the destruction of adrenal cortex layers and hormone deficiencies.^7^ One of the most common risk factors for BAH is APS, a condition known to cause thrombosis and hemorrhage.^8^ Although the exact cause remains unclear, bacterial infections, coagulopathy, and disseminated intravascular coagulation are thought to play key roles in the development of AH, with anticoagulant use, particularly heparin exposure, being a commonly reported risk factor.^9^ Although adrenal neoplasms are known to cause AH, they rarely present as the primary condition at first. A systematic review of 133 patients with AH found that adrenal neoplasms, although a recognized cause, rarely present initially as such. Of these, 48% were pheochromocytomas, 14% were metastases, and 13% were hematomas masquerading as neoplasms.^4^ Our patient lacked common risk factors for BAH, such as trauma, anticoagulation therapy, APS, or sepsis, making the underlying cause of AH initially unclear.

The diagnosis of BL requires adequate tissue sampling, with excisional biopsy preferred over fine-needle aspiration. Microscopy, flow cytometry, immunohistochemistry, and cytogenetics confirm the diagnosis.^5^ The management of BL requires immediate initiation of treatment, ideally within 48 hours of diagnosis, due to its highly aggressive nature. In pediatric and young adult populations, the overall cure rate approaches 90% in developed countries.^5^ For pediatric patients, it depends on the extent of the disease. Two cycles of moderate-intensity chemotherapy (eg, *cyclophosphamide*, *vincristine*, *prednisolone*, and *doxorubicin*) are typically sufficient.^5^ Supportive care is essential throughout treatment, particularly for managing tumor lysis syndrome.^10^ For BL with adrenal involvement, treatment should involve adrenal hormone replacement.^7^

CT imaging is the preferred modality for detecting AH, whereas magnetic resonance imaging provides the highest diagnostic accuracy, allowing for differentiation between acute and chronic hematomas.^1^ According to the literature, we should promptly assess the adrenal function in cases of suspected AH due to the risk of AI. This includes evaluating plasma electrolytes and basal adrenal hormone levels. An adrenocorticotropic hormone stimulation test is reserved for unclear basal hormone results or follow-up to monitor adrenal recovery if AI is diagnosed.^7^ Management of AH depends on the extent of hemorrhage and the presence of AI. Conservative treatment with serial imaging and close monitoring is often preferred, particularly in hemodynamically stable patients. However, massive retroperitoneal bleeding that is unresponsive to transfusions may require intervention.^11^ Angiography with embolization is favored over surgical laparotomy for better outcomes. In cases of AI due to extensive BAH, corticosteroid replacement is essential to prevent life-threatening adrenal failure. Early recognition of adrenal congestion on CT scan can aid in timely steroid initiation. Pre-emptive steroid use in high-risk patients may reduce mortality from adrenal crisis.^11^

A literature review identified a very few instances of AI co-occurring with lymphoma. The first and only reported case of spontaneous unilateral AH occurred in a 56-year-old previously healthy woman, who was diagnosed with primary adrenal diffuse large B-cell lymphoma.^12^ Another case of bilateral adrenal masses without AH in an 85-year-old woman caused AI and was ultimately diagnosed as a high-grade non-Hodgkin lymphoma with intermediate features between diffuse large B-cell lymphoma and BL, characterized by a high proliferation index (Ki-67 > 90%).^13^ Most of these cases were treated with rituximab, cyclophosphamide, hydroxydaunomycin, oncovin and prednisone chemotherapy, which significantly reduced mass size and resulted in complete remission.

## Conclusion

In conclusion, this case highlights the importance of considering malignancy in patients presenting with unexplained AH and AI, even in the absence of conventional risk factors. Future research should explore the underlying mechanisms linking BL and AH. Unlike typical cases of BL, our patient had an AH as an early and predominant feature. This highlights the need for a broad differential diagnosis in patients with AH, particularly when AI develops.

## Disclosure

The authors have no conflicts of interest to disclose.
